# Relationship between dietary characteristics and heel quantitative ultrasound parameters in postmenopausal women from the OsteoLaus cohort

**DOI:** 10.1038/s41598-024-51774-5

**Published:** 2024-01-18

**Authors:** A. Lanyan, P. Marques-Vidal, A. Métrailler, E. Gonzalez Rodriguez, D. Hans, E. Shevroja, O. Lamy

**Affiliations:** 1 Bone Diseases CENTER, Lausanne University Hospital (CHUV), Rue du Bugnon 46, 1011 Lausanne, Switzerland; 2 Service of Internal Medicine, Lausanne University Hospital and University of Lausanne, Rue du Bugnon 46, 1011 Lausanne, Switzerland

**Keywords:** Bone, Osteoporosis, Bone, Osteoporosis

## Abstract

The role of dietary patterns in the development of osteoporosis is unclear. The heel quantitative ultrasound (QUS) is a potential alternative to Dual X-Ray Absorptiometry. Nutrients, foods, dietary patterns and compliance to dietary guidelines were compared between the lowest and the highest tertiles of QUS parameters [Broadband Ultrasound Attenuation (BUA), Speed of Sound (SOS), Stiffness Index (SI)], using data from the OsteoLaus cohort. Participants in the highest tertiles of QUS parameters (385 for BUA, 397 for SOS, 386 for SI) were younger, of higher body weight, and had less major osteoporotic fractures. Women in the highest tertiles of SI and BUA consumed more fat (35.1 ± 0.4 vs 33.9 ± 0.4 and 34.9 ± 0.4 vs 33.8 ± 0.4 gr/day for SI and BUA, respectively, *p* < 0.05), and complied less frequently with dairy intake guidelines [odds ratio (95% confidence interval): 0.70 (0.53–0.92) and 0.72 (0.55–0.95) for SI and BUA, respectively, *p* < 0.05] than women in the lowest tertile. No differences were found regarding dietary patterns, healthy dietary scores, or compliance to dietary guidelines. Postmenopausal women in the highest QUS tertiles were younger, of higher weight and BMI, consumed more monounsaturated fatty acids and less dairy and calcium than women in the lowest tertiles. No differences were found between QUS tertiles regarding dietary patterns.

## Introduction

Osteoporosis, the combination of reduced bone density and altered microarchitecture, leads to increased fracture risk and represents a severe clinical and socio-economic burden in our ageing population countries^1^. A recent literature review showed that the yearly cost of osteoporosis-related prevalent and incident fragility fractures was 3430 million euros in Switzerland in 2019, and corresponded to a loss of 5166 million euros in quality-adjusted life years (QALYs)^2^. The QALYs accounted for severe pain, physical disability, loss of autonomy, medical complications and even death in the concerned population.

The growing burden of osteoporosis warrants the necessity to develop efficient screening tests for osteoporosis and fracture risk. As of the diagnosis of osteoporosis, Dual-energy X-ray absorptiometry (DXA) remains the gold standard^3^. Yet, DXA is an expensive and constraining exam, which is limited to specialized centers. Quantitative ultrasound (QUS) is a potential alternative to DXA. It is a peripheral bone measure technique, the clinical utility of which has mainly been demonstrated when performed on the heel bone, the calcaneum^4^. As advantages over DXA, QUS does not emit radiation, is lower cost and has quicker accessibility and wider availability^5^.

Moreover, some studies have shown that bone status should not only be evaluated through its quantity (mostly assessed by DXA), but also through its quality^6^, a feature that can be assessed using QUS^7^. The main measured QUS parameters are Broadband Ultrasound Attenuation (BUA), Speed of Sound (SOS), and Stiffness Index (SI)^8^. A porous trabecular bone attenuates less and is slower crossed by the ultrasound.

In terms of modifiable risk factors for osteoporosis, diet has broadly been studied and remains a possible lever for its prevention^9^. From the European ESCEO guidelines^10^, the American Association of Clinical Endocrinologists Guidelines^11^, to the recent Swiss recommendations^12^, all mention calcium and vitamin D adequate consumption or supplementation. Yet, besides nutrients and foods, dietary habits are assessed more thoroughly through patterns and scores, as it is crucial to consider diet as a combination of different foods and not an intake of isolated micronutrients. Studies show a higher benefit of the Mediterranean diet and of patterns that emphasized the intake of fruits, vegetables, whole grains, poultry and fish, nuts and legumes and low-fat dairy products, and de-emphasized the intake of soft drinks, fried foods, meat and processed products, sweets and desserts and refined grains^13^. Whether these well-established strategies have impact on bone health as assessed by QUS remains an arising question, although relevant evidence^14,15^ exist.

We therefore evaluated the associations between dietary intake (as assessed by foods and nutrient intake) and quality (as assessed by dietary patterns, dietary scores, and compliance to the Swiss Society of Nutrition (SSN) guidelines) and bone QUS measurements among postmenopausal women in the Lausanne OsteoLaus Cohort.

## Methods

### Participants

The OsteoLaus Study is a substudy of the CoLaus|PsyCoLaus study, an ongoing population-based prospective study aiming to assess the determinants of cardiovascular and psychiatric diseases in the citizens of Lausanne, Switzerland^16^. The aim of OsteoLaus is to obtain more precise fracture risk models and to evaluate the link between cardiovascular diseases and osteoporosis^17^. Between September 2009 and September 2012, all women aged between 50 and 80 years from the CoLaus study were invited to participate in OsteoLaus. Of the initial 1704 women invited, 1500 (88%) accepted, and 1475 were included; 98.4% of which were Caucasian^17^. OsteoLaus women have undergone a follow-up visit with thorough bone health assessment every 2.5 years and are currently undergoing the fifth and last study visit. As QUS measurement was performed only at the baseline (and the fifth study visit, for which the data is not yet available), participating women in the baseline visit were included in this analysis.

### Quantitative ultrasound assessment

Heel QUS measures were performed using the Achilles Express apparatus (GEHC Lunar Co., Madison, WI, USA)*.* Daily quality control was performed in accordance with the manufacturer’s recommendations. All measurements were done by the same operator and performed on the right heel. The measurements were performed on the left heel if the participant had history of a previous fracture in the right lower extremity. Two transducers (a transmitting and a receiving one) were positioned at each side of the heel. Two parameters were generated by QUS: BUA, the slope of the sound wave attenuation depending on its frequency, given in dB/MHz; the Speed of Sound (SOS), the distance between the two transducers divided by the time it took for the signal to pass from one to the other, given in m/sec; and the SI, calculated automatically from the device through this formula: SI = (0.67 × BUA) + (0.28 × SOS) – 420, unitless.

### Dietary intake

Dietary intake was assessed in CoLaus using a validated, self-administered, semi-quantitative food frequency questionnaire (FFQ) which also included portion size^18^. Briefly, this FFQ assesses the dietary intake of the previous 4 weeks and consists of 97 different food items, which can be grouped in different food categories: dairy products, meat (including red meat, processed meat, and white meat), fish (including shellfish), wholegrain products, fruits, vegetables, others (containing pasta, ravioli, rice, tomato sauce, couscous, pizza, quiche, eggs and tofu), spreads and sauces, fruits, pastries and sweets, cooking oils, vitamins and supplements, and drinks. For this study, we considered dairy products, red meat, processed meat, wholegrain products, fruits, vegetables, and fish. Consumption frequencies ranged from “less than once during the last 4 weeks” to “2 or more times per day”, and participants indicated the average serving size (smaller, equal, or bigger) compared with a reference size.

Reported frequencies were transformed into daily consumption frequencies as follows: “never these last 4 weeks” = 0, “once/month” = 1/28, “2–3/month” = 2.5/28, “1–2/week” = 1.5/7, “3–4 times/week” = 3.5/7, “once/day” = 1 and “2 + / day” = 2.5. The consumption frequency of one food category was obtained by summing up all individual consumption frequencies of foods related to that category^19^. For example, daily fruit consumption was obtained by summing up the daily consumptions of fresh fruits (five items). For each food, daily frequencies were multiplied by the average serving size to obtain the amount of the food consumed per day; this amount was used to compute the contribution of the selected food to total energy, macronutrient, and micronutrient intake, using the French CIQUAL food composition table (latest version available at https://ciqual.anses.fr/). Macronutrients were reported as percentage of total energy intake (TEI). Due to the great variability of dosages^20^, calcium and vitamin D intake from supplements was not considered. Average amounts consumed per day were computed for dairy products, red meat, processed meat, wholegrain products, fruits (excluding canned and fruit juices), vegetables and fish (all and excluding fried fish).

The quality of dietary intake was assessed using three different approaches. The first approach assessed dietary quality via three dietary scores: (i) Mediterranean score 1^21^ ranges between 0 and 8; (ii) Mediterranean score 2, adapted to the Swiss population, ranges between 0 and 9^22^; contrary to Mediterranean score 1, in Mediterranean score 2, dairy products are considered as beneficial; and iii) the alternative healthy eating index (AHEI)^23^; which does not include dairy products. In our study, the amount of *trans* fatty acids could not be assessed, and we considered all participants taking multivitamins as taking them for a duration ≥ 5 years. This last assumption was taken as no information was available regarding duration of multivitamin use. Thus, the modified AHEI score ranged between 2.5 and 77.5 instead of 2.5 and 87.5 for the original one^23^. For all three scores, higher values represented a healthier diet.

The second approach assessed dietary quality via dietary patterns, assessed using consumption frequencies as reported previously^24^. Briefly, principal component analysis was applied, and three “naive” dietary patterns were obtained: (i) “meat and chips,” with high loadings for all types of meat and French fries; (ii) “fruits and vegetables,” with high loadings for most fruits and vegetables; and (iii) “fatty and sugary,” with high loadings for fatty and sweet foods^25^.

The third approach assessed dietary quality via the compliance to the SSN for fruits, vegetables, meat, fish and dairy products^25^. The guidelines are (a) ≥ 2 fruit portions/day, (b) ≥ 3 vegetable portions/day, (c) ≤ 5 meat portions/week, (d) ≥ 1 fish portion/week and (e) ≥ 3 dairy product portions/day. As the FFQ queried about fresh and fried fish, two categories were considered: one including and one excluding fried fish, as several studies have shown that fried fish or fried foods are associated with an increased risk of cardiovascular events^26^. Participants were further dichotomized if they complied with at least three guidelines or not; two categories of compliance were created, depending on the type of fish consumed (including or excluding fried fish).

### Covariates

Each participant had a questionnaire on potential risk factors for fracture or osteoporosis, on conditions affecting bone metabolism and on prevalent fractures.

Participants were queried regarding their medical treatment, physical activity, and socio-economic status. Educational level was self-reported using a questionnaire and categorized into low (apprenticeship or mandatory), middle (high school) and high (university) education. Smoking status was self-reported and categorized into never, former (irrespective of the time since quitting) and current (irrespective of the amount of tobacco smoked). Physical activity was assessed using a physical activity frequency questionnaire (PAFQ) validated in the population of Geneva, Switzerland^27^. Sedentary status was considered if the participant spent less than 10% of daily time in activities ≥ 4 times the basal metabolic rate^28,29^.

Body weight and height were measured with participants’ barefoot and in light indoor clothes. Body weight was measured in kilograms to the nearest 100 g using a Seca scale (Hamburg, Germany). Height was measured to the nearest 5 mm using a Seca (Hamburg, Germany) height gauge. Body mass index (BMI) was categorized into normal + low (< 25 kg/m^2^), overweight (25–29.99 kg/m^2^) and obese (≥ 30 kg/m^2^).

Blood was drawn in the morning after overnight fasting to diagnose diabetes. Biological assays were performed by the CHUV Clinical Laboratory on fresh blood samples within 2 h of blood collection. Glucose levels were assessed using glucose hexokinase, with maximum inter- and intra-batch coefficient of variation (CVs) of 1.6% and 0.8%, respectively. Diabetes was considered for a fasting plasma glucose level ≥ 7.0 mmol/l or the intake of antidiabetic treatment. Although several measurements are recommended to diagnose diabetes, this would be impractical to perform in an epidemiological setting.

Major osteoporotic fractures included at least one fracture of the vertebrae (clinical or radiologic from grade 2/3 on vertebral fracture assessment (VFA), hip, pelvis, humerus, and radius, occurring spontaneously or after falling from the participant’s own height.

### Exclusion criteria

OsteoLaus baseline participants were excluded from this analysis if they (1) had no data for dietary intake or QUS parameters, (2) reported a total energy intake < 500 or > 3500 cal/day or (3) had missing data for covariates.

### Statistical analysis

Statistical analysis was performed using the Stata version 15.0 for Windows (Stata Corp., College Station, TX, USA). Descriptive results were expressed as the number of participants (percentage) for categorical variables or as average ± standard deviation or median [interquartile range] for continuous variables. Distribution of the variables was assessed by visually inspecting the histograms and QQ plots. Bivariate comparisons between groups were performed using chi-squared or Fisher’s exact test for categorical variables; and Student’s t test, analysis of variance or Kruskal–Wallis test for continuous variables. Associations between bone QUS measures and dietary markers were assessed using Spearman correlation.

Macronutrient intake was assessed using the energy density method, i.e., expressed as percentage of the TEI. A second analysis was conducted using the energy regression method as suggested by Willett et al.^30^. Briefly, the amounts of macronutrients were regressed on TEI and the resulting residuals were compared between groups.

Multivariable analysis was performed using logistic regression for categorical variables and analysis of variance (ANOVA) for continuous variables. The highest and lowest tertiles of QUS parameters were used as categories when applying ANOVA and as independent variables when using logistic regression. As QUS measurements do not have internationally approved cut-offs, we decided to work with tertiles focusing our analyses on the lowest and the highest QUS parameters values: (1) SI (2) BUA (3) SOS. Multivariable models were adjusted for total energy intake (continuous), age (continuous), BMI (continuous), educational level (four categories: mandatory/apprenticeship/high school/university), antiosteoporotic treatment (yes/no), sedentary status (yes/no) or diabetes (yes/no). Results were expressed as multivariable adjusted odds ratio (OR) and 95% confidence interval (95% CI) for the logistic models and as multivariable adjusted average ± standard error for analysis of variance.

Statistical significance was assessed for a two-sided test with *p* < 0.05. No correction for multiple testing was applied as our study was exploratory and we wanted to identify the largest number of dietary markers potentially associated with bone ultrasound parameters^31^.

### Ethical statement

The CoLaus and OsteoLaus studies were approved by the Institutional Ethics Committee of the University of Lausanne, which afterwards became the Ethics Commission of Canton Vaud (http://www.cer-vd.ch ). The studies were performed in agreement with the Helsinki Declaration and its former amendments and in accordance with the applicable Swiss legislation. All participants gave their written informed consent before entering the study.

## Results

### Characteristics of participants

**Figure 1:**
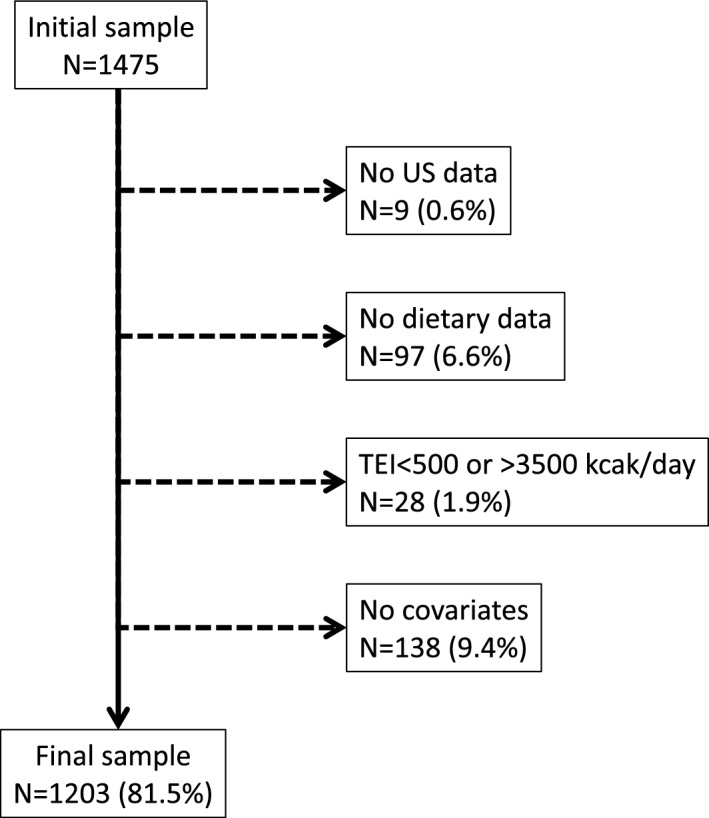
Flow chart, selection of participants, OsteoLaus study, Lausanne, Switzerland.

From the initial 1475 women, 272 (18.5%) were excluded. A flowchart of the study population is presented in Fig. 1. The characteristics of the included and excluded participants are provided in Supplementary table 1. Excluded participants were significantly older (*p* = 0.004), had a higher BMI (*p* < 0.001) and presented more frequently with diabetes (*p* < 0.001).

The characteristics of the included participants according to lowest and highest tertiles of QUS parameters are summarized in Table 1. At baseline, for the whole cohort, the mean ± SD values were SOS (m/s) 1547.9 ± 32.0; BUA (dB/MHz) 107.9 ± 13.8; SI (unitless) 85.2 ± 15.9. The values of the tertiles were < 1533 m/s (lowest) and > 1560 m/s (highest) for SOS, < 102 dB/MHz (lowest) and > 113 dB/MHz (highest) for BUA, < 78 (lowest), > 91 (highest) for SI.

**Table 1:** Characteristics of the participants in the lowest and highest tertiles of QUS parameters, OsteoLaus study, Lausanne, Switzerland.

	Stiffness Index			Broadband US attenuation			Speed of sound		
	Lowest tertile	Highest tertile	*P*-value	Lowest tertile	Highest tertile	*P*-value	Lowest tertile	Highest tertile	*P*-value
Sample size	409	386		413	385		402	397	
Age (years)	66.8 ± 6.9	61.8 ± 7.3	< 0.001	66.9 ± 6.9	62.1 ± 7.2	< 0.001	66.4 ± 6.9	62.5 ± 7.8	< 0.001
Age groups (%)			< 0.001			< 0.001			< 0.001
50–59	64 (15.7)	170 (44.0)		63 (15.3)	161 (41.8)		73 (18.2)	163 (41.1)	
60–69	215 (52.6)	159 (41.2)		215 (52.1)	168 (43.6)		202 (50.3)	160 (40.3)	
70–79	130 (31.8)	57 (14.8)		135 (32.7)	56 (14.6)		127 (31.6)	74 (18.6)	
Education (%)			0.006			0.008			0.020
High	47 (11.5)	69 (17.9)		45 (10.8)	67 (17.4)		47 (11.7)	69 (17.3)	
Middle	103 (25.2)	112 (29.0)		108 (26.2)	112 (29.1)		104 (25.9)	115 (29.0)	
Low	259 (63.3)	205 (53.1)		260 (63.0)	206 (53.5)		251 (62.4)	213 (53.7)	
Weight (kg)	65.2 ± 11.7	69.5 ± 12.2	< 0.001	64.2 ± 11.4	71.2 ± 12.1	< 0.001	67.2 ± 11.8	67.5 ± 11.9	0.697
BMI (kg/m^2^)	25.1 ± 4.4	26.4 ± 4.4	< 0.001	25.0 ± 4.5	26.8 ± 4.4	< 0.001	25.6 ± 4.3	26.0 ± 4.5	0.207
BMI categories (%)			< 0.001			< 0.001			0.342
Normal	228 (55.8)	152 (39.4)		231 (55.9)	136 (35.3)		198 (49.3)	175 (44.1)	
Overweight	114 (27.9)	161 (41.7)		110 (26.6)	174 (45.2)		139 (34.6)	151 (38.0)	
Obese	67 (16.4)	73 (18.9)		72 (17.4)	75 (19.5)		65 (16.2)	71 (17.9)	
Smoking status (%)			0.001			0.008			< 0.001
Never	193 (47.2)	167 (43.3)		204 (49.4)	165 (42.9)		185 (46.0)	185 (46.6)	
Former	131 (32.0)	168 (43.5)		142 (34.4)	173 (44.9)		130 (32.3)	165 (41.6)	
Current	85 (20.8)	51 (13.2)		67 (16.2)	47 (12.2)		87 (21.6)	47 (11.8)	
Physical activity: sedentary (%)	272 (66.5)	246 (63.7)	0.412	270 (65.4)	242 (62.9)	0.459	279 (69.4)	245 (61.7)	0.022
Participants with diabetes (%)	24 (5.9)	32 (8.3)	0.182	24 (5.8)	34 (8.8)	0.101	24 (6.0)	32 (8.1)	0.247
Major fractures (%)	73 (17.9)	13 (3.4)	< 0.001	69 (16.7)	14 (3.6)	< 0.001	64 (15.9)	19 (4.8)	< 0.001

BMI, body mass index; US, ultrasound. Results are expressed as number of participants (percentage) for categorical variables or as average ± standard deviation for continuous variables. Between group comparisons using chi-square for categorical variables or student’s t-test for continuous variables.

Participants in the highest tertiles of SI, BUA and SOS were significantly younger (*p* < 0.001), more highly educated (*p* < 0.05), more frequently never smokers (*p* < 0.01) and had fewer major fractures (*p* < 0.001). Participants in the highest tertiles of SI and BUA also had significantly higher weight and BMI (both *p* < 0.001).

### Associations between bone ultrasound markers and diet, bivariate analysis

The comparison of the dietary markers between the highest and the lowest tertiles of QUS are presented in Table 2. Participants in the highest tertiles of SI, BUA and SOS consumed significantly higheramounts of monounsaturated fatty acids (MUFA) (*p* < 0.05), had a lower score of the “Fatty and sugary” dietary pattern (*p* < 0.05) and complied less to dairy SSN guidelines (*p* < 0.005) than participants in the lowest tertiles. Participants in the highest tertiles of SI and BUA also consumed significantly lower amounts of carbohydrates (*p* < 0.005) but higher amounts of total fats (*p* = 0.002), than participants in the lowest tertiles. Participants in the highest tertile of SI consumed less calcium (*p* < 0.05), lower amounts of fruits (*p* = 0.025), and complied less to the fruits (*p* = 0.043), and to at least three dietary guidelines – including all types of fish—(*p* = 0.005), than participants in the lowest tertile.

**Table 2:** Bivariate comparison of the dietary markers between the lowest and highest tertiles of QUS parameters, OsteoLaus study, Lausanne, Switzerland.

	Stiffness Index			Broadband US Attenuation			Speed of Sound		
	Lowest tertile	Highest tertile	*P*-value	Lowest tertile	Highest tertile	*P*-value	Lowest tertile	Highest tertile	*P*-value
Sample size	409	386		413	385		402	397	
Total energy intake (kcal)	1621 [1257–2029]	1510 [1211–1926]	§ 0.033	1626 [1240–2040]	1515 [1207–1941]	§ 0.069	1623 [1240–2063]	1530 [1203–1951]	§ 0.102
Nutrients (% TEI)									
Protein	15.2 ± 2.9	15.5 ± 3.4	0.167	15.1 ± 3.1	15.6 ± 3.5	0.055	15.2 ± 2.9	15.3 ± 3.1	0.724
Vegetal protein	4.8 ± 1.3	4.7 ± 1.2	0.208	4.8 ± 1.3	4.6 ± 1.1	0.023	4.8 ± 1.2	4.7 ± 1.2	0.504
Animal protein	10.4 ± 3.3	10.8 ± 3.8	0.097	10.3 ± 3.5	10.9 ± 3.9	0.014	10.4 ± 3.2	10.6 ± 3.4	0.594
Total carbohydrates	48.1 ± 8.8	46.4 ± 8.9	0.005	48.3 ± 8.5	46.4 ± 9.2	0.002	47.8 ± 8.9	47.0 ± 8.8	0.210
Monosaccharides	25.8 ± 8.8	24.6 ± 8.6	0.052	25.8 ± 8.4	24.8 ± 8.8	0.132	25.6 ± 8.8	25.2 ± 8.8	0.495
Polysaccharides	22.3 ± 7.9	21.7 ± 7.3	0.287	22.5 ± 8.0	21.4 ± 7.5	0.056	22.1 ± 7.7	21.7 ± 7.2	0.484
Total fat	33.7 ± 7.1	35.3 ± 7.0	0.002	33.6 ± 6.8	35.1 ± 7.0	0.002	34.0 ± 7.1	34.8 ± 7.0	0.098
SFA	12.3 ± 3.3	12.6 ± 3.3	0.224	12.3 ± 3.3	12.6 ± 3.4	0.208	12.4 ± 3.2	12.4 ± 3.2	0.976
MUFA	13.5 ± 3.8	14.5 ± 4.0	< 0.001	13.5 ± 3.6	14.4 ± 3.9	< 0.001	13.7 ± 3.8	14.3 ± 4.0	0.015
PUFA	4.7 ± 1.5	4.9 ± 1.5	0.019	4.7 ± 1.4	4.9 ± 1.5	0.024	4.8 ± 1.7	4.9 ± 1.5	0.206
Alcohol	3.0 ± 5.1	2.9 ± 4.0	0.756	2.9 ± 4.8	2.9 ± 4.1	0.922	3.1 ± 5.2	2.9 ± 4.2	0.737
Micronutrients									
Calcium (mg/day)	921 [646–1318]	863 [635–1142]	§ 0.036	899 [646–1303]	868 [647–1163]	§ 0.244	920 [637–1318]	869 [652–1214]	§ 0.124
Vitamin D (μg/day)	2.0 [1.2–2.8]	2.2 [1.3–3.1]	§ 0.216	2.0 [1.2–2.9]	2.2 [1.2–3.1]	§ 0.181	2.0 [1.2–2.9]	2.1 [1.2–3.1]	§ 0.513
Food items (g/day)									
Dairy products	172 [87–287]	158 [93–251]	§ 0.331	176 [92–287]	164 [88–261]	§ 0.312	176 [85–276]	159 [90–258]	§ 0.327
Red meat	27 [14–48]	28 [15–51]	§ 0.156	26 [14–48]	29 [15–50]	§ 0.099	28 [15–48]	28 [15–53]	§ 0.325
Processed meat	7 [2–13]	7 [2–13]	§ 0.861	7 [2–12]	7 [2–13]	§ 0.809	6 [3–13]	7 [2–13]	§ 0.802
Wholegrain products	38 [11–75]	38 [11–68]	§ 0.614	41 [11–77]	36 [11–66]	§ 0.109	38 [11–75]	38 [11–75]	§ 0.805
Fruits	248 [130–403]	204 [110–375]	§ 0.043	248 [136–403]	220 [115–390]	§ 0.225	254 [130–408]	213 [115–398]	§ 0.135
Vegetables	138 [95–221]	143 [103–214]	§ 0.298	139 [95–208]	142 [103–210]	§ 0.290	139 [92–214]	151 [105–219]	§ 0.062
Fish (all)	24 [13–41]	27 [13–43]	§ 0.412	23 [11–41]	27 [13–42]	§ 0.194	23 [11–39]	26 [11–44]	§ 0.104
Fish (excluding fried)	31 [19–47]	32 [17–50]	§ 0.789	29 [16–48]	31 [18–50]	§ 0.492	29 [17–46]	32 [17–50]	§ 0.280
Hypothesis-oriented dietary scores									
Mediterranean score 1	4 [3–5]	4 [3–5]	§ 0.828	4 [3–5]	4 [3–5]	§ 0.089	4 [3–5]	4 [3–5]	§ 0.247
Mediterranean score 2	5 [3–6]	5 [3–6]	§ 0.475	5 [3–6]	5 [3–6]	§ 0.881	5 [3–6]	5 [3–6]	§ 0.076
AHEI	33.5 ± 10.1	33.2 ± 10.3	0.652	33.8 ± 10.3	33.5 ± 10.2	0.660	33.3 ± 10	33.7 ± 10.4	0.560
Naïve dietary scores									
Meat & chips	− 0.45 ± 1.04	− 0.38 ± 0.94	0.380	− 0.45 ± 1.07	− 0.35 ± 1.00	0.189	− 0.45 ± 1.15	− 0.38 ± 1.00	0.386
Fruits & vegetables	0.42 ± 1.56	0.49 ± 1.62	0.566	0.47 ± 1.56	0.45 ± 1.57	0.852	0.39 ± 1.56	0.54 ± 1.61	0.199
Fatty and sugary	− 0.02 ± 1.38	− 0.26 ± 1.30	0.015	0.02 ± 1.41	− 0.21 ± 1.34	0.021	− 0.01 ± 1.40	− 0.21 ± 1.37	0.046
Compliance to dietary Swiss guidelines									
Fruits ≥ 2/day	236 (57.7)	186 (48.2)	0.007	236 (57.1)	194 (50.4)	0.056	229 (57.0)	204 (51.4)	0.113
Vegetables ≥ 3/day	36 (8.8)	36 (9.3)	0.797	35 (8.5)	30 (7.8)	0.725	37 (9.2)	37 (9.3)	0.955
Meat ≤ 5/week	297 (72.6)	268 (69.4)	0.322	305 (73.9)	264 (68.6)	0.100	287 (71.4)	277 (69.8)	0.615
Fish ≥ 2/week	273 (66.8)	258 (66.8)	0.978	266 (64.4)	261 (67.8)	0.313	255 (63.4)	267 (67.3)	0.256
Fish (excl. fried) ≥ 2/week	178 (43.5)	193 (50.0)	0.067	178 (43.1)	191 (49.6)	0.065	169 (42.0)	196 (49.4)	0.038
Dairy ≥ 3/day	53 (13.0)	23 (6.0)	0.001	54 (13.1)	25 (6.5)	0.002	50 (12.4)	26 (6.6)	0.005
At least three guidelines †	152 (37.2)	108 (28.0)	0.006	158 (38.3)	111 (28.8)	0.005	139 (34.6)	122 (30.7)	0.246
At least three guidelines ††	123 (30.1)	90 (23.3)	0.032	127 (30.8)	91 (23.6)	0.024	114 (28.4)	96 (24.2)	0.180

^†^, all fish; ††, excluding fried fish. AHEI, alternative healthy eating index; MUFA, monounsaturated fatty acids; PUFA, polyunsaturated fatty acids; SFA, saturated fatty acids; TEI, total energy intake; US, ultrasound. Results are expressed as number of participants (percentage) for categorical variables and as average ± standard deviation or median [interquartile range] for continuous variables. Between group comparisons using chi-square for categorical variables and student’s t-test or Kruskal–Wallis test (§) for continuous variables.

### Associations between bone ultrasound markers and diet, multivariable analysis

The results of the associations between tertiles of QUS and dietary markers after multivariable adjustment are presented in Table 3. Participants in the highest tertiles of SI, BUA and SOS had a lower likelihood of complying to the dairy SSN guideline (*p* < 0.05) than participants in the lowest tertiles. Participants in the highest tertiles of SI and BUA had a significantly higher consumption of total fat (*p* < 0.05) in particular MUFA (*p* < 0.05), and had a lower likelihood of complying to at least three SSN dietary guidelines (*p* < 0.05) than participants in the lowest tertiles. Participants in the highest tertiles of SI and SOS also had a significantly lower calcium consumption (*p* < 0.05).

**Table 3:** Multivariable analysis of the dietary markers among participants with lowest or highest tertiles of QUS parameters, OsteoLaus study, Lausanne, Switzerland.

	Stiffness Index			Broadband US attenuation			Speed of sound		
	Low tertile	High tertile	*P*-value	Low tertile	High tertile	*P*-value	Low tertile	High tertile	*P*-value
Sample size	409	386		413	385		402	397	
Nutrients (% TEI)									
Protein	15.3 ± 0.2	15.4 ± 0.2	0.839	15.3 ± 0.2	15.4 ± 0.2	0.963	15.3 ± 0.2	15.2 ± 0.2	0.880
Vegetal protein	4.8 ± 0.1	4.7 ± 0.1	0.336	4.8 ± 0.1	4.7 ± 0.1	0.125	4.8 ± 0.1	4.7 ± 0.1	0.278
Animal protein	10.5 ± 0.2	10.6 ± 0.2	0.628	10.5 ± 0.2	10.7 ± 0.2	0.402	10.5 ± 0.2	10.5 ± 0.2	0.829
Total carbohydrates	47.9 ± 0.5	46.7 ± 0.5	0.082	48.0 ± 0.5	46.8 ± 0.5	0.093	47.7 ± 0.4	47.0 ± 0.5	0.308
Monosaccharides	25.6 ± 0.5	24.7 ± 0.5	0.202	25.6 ± 0.4	25.0 ± 0.5	0.340	25.5 ± 0.5	25.3 ± 0.5	0.755
Polysaccharides	22.2 ± 0.4	21.8 ± 0.4	0.566	22.2 ± 0.4	21.7 ± 0.4	0.390	22.2 ± 0.4	21.7 ± 0.4	0.393
Total fat	33.9 ± 0.4	35.1 ± 0.4	0.025	33.8 ± 0.4	34.9 ± 0.4	0.047	34.0 ± 0.4	34.7 ± 0.4	0.166
SFA	12.3 ± 0.2	12.6 ± 0.2	0.175	12.3 ± 0.2	12.6 ± 0.2	0.251	12.3 ± 0.2	12.4 ± 0.2	0.602
MUFA	13.7 ± 0.2	14.4 ± 0.2	0.022	13.7 ± 0.2	14.3 ± 0.2	0.042	13.8 ± 0.2	14.2 ± 0.2	0.096
PUFA	4.7 ± 0.1	4.9 ± 0.1	0.094	4.7 ± 0.1	4.9 ± 0.1	0.140	4.8 ± 0.1	4.9 ± 0.1	0.234
Alcohol	3.0 ± 0.2	2.9 ± 0.2	0.808	2.9 ± 0.2	2.9 ± 0.2	0.977	3.0 ± 0.2	3.0 ± 0.2	0.947
Micronutrients									
Calcium (mg/day)	1030 ± 24	938 ± 25	0.012	1015 ± 25	976 ± 26	0.311	1032 ± 24	951 ± 24	0.023
Vitamin D (μg/day)	2.4 ± 0.1	2.5 ± 0.1	0.303	2.4 ± 0.1	2.5 ± 0.1	0.579	2.4 ± 0.1	2.5 ± 0.1	0.531
Food items (g/day)									
Dairy products	217 ± 9	199 ± 10	0.172	221 ± 9	206 ± 10	0.278	217 ± 9	201 ± 9	0.220
Red meat	35 ± 2	36 ± 2	0.652	35 ± 2	36 ± 2	0.482	36 ± 2	37 ± 2	0.692
Processed meat	10 ± 1	10 ± 1	0.731	9 ± 1	9 ± 1	0.852	10 ± 1	10 ± 1	0.968
Wholegrain products	53 ± 3	51 ± 3	0.705	53 ± 3	52 ± 3	0.791	53 ± 3	52 ± 3	0.688
Fruits	304 ± 14	285 ± 14	0.353	304 ± 13	290 ± 14	0.511	308 ± 14	295 ± 14	0.401
Vegetables	170 ± 6	174 ± 6	0.644	167 ± 6	170 ± 6	0.714	169 ± 5	173 ± 6	0.659
Fish (all)	36 ± 2	36 ± 2	0.966	37 ± 2	36 ± 2	0.875	35 ± 2	37 ± 2	0.455
Fish (excluding fried)	30 ± 1	30 ± 1	0.888	30 ± 1	30 ± 1	0.959	29 ± 1	31 ± 1	0.424
Hypothesis-oriented dietary scores									
Mediterranean score 1	3.9 ± 0.1	3.9 ± 0.1	0.962	4.0 ± 0.1	3.8 ± 0.1	0.083	3.9 ± 0.1	4.0 ± 0.1	0.603
Mediterranean score 2	4.7 ± 0.1	4.6 ± 0.1	0.884	4.8 ± 0.1	4.7 ± 0.1	0.540	4.7 ± 0.1	4.7 ± 0.1	0.941
AHEI	34 ± 1	33 ± 1	0.193	34 ± 1	33 ± 1	0.371	34 ± 1	33 ± 1	0.594
Naïve dietary scores									
Meat & chips	− 0.4 ± 0.1	− 0.4 ± 0.1	0.988	− 0.4 ± 0.1	− 0.4 ± 0.1	0.857	− 0.4 ± 0.1	− 0.4 ± 0.1	0.774
Fruits & vegetables	0.4 ± 0.1	0.5 ± 0.1	0.645	0.5 ± 0.1	0.5 ± 0.1	0.933	0.4 ± 0.1	0.5 ± 0.1	0.531
Fatty and sugary	− 0.1 ± 0.1	− 0.2 ± 0.1	0.196	− 0.1 ± 0.1	− 0.1 ± 0.1	0.503	0 ± 0.1	− 0.2 ± 0.1	0.085
Compliance to dietary Swiss guidelines									
Fruits ≥ 2/day	1 (ref.)	0.86 (0.74–1.00)	0.053	1 (ref.)	0.90 (0.77–1.06)	0.198	1 (ref.)	0.88 (0.76–1.02)	0.090
Vegetables ≥ 3/day	1 (ref.)	1.04 (0.79–1.35)	0.793	1 (ref.)	0.98 (0.74–1.31)	0.909	1 (ref.)	0.95 (0.74–1.22)	0.683
Meat ≤ 5/week	1 (ref.)	1.01 (0.85–1.19)	0.929	1 (ref.)	0.95 (0.80–1.12)	0.527	1 (ref.)	1.01 (0.86–1.19)	0.897
Fish ≥ 2/week	1 (ref.)	0.89 (0.76–1.05)	0.177	1 (ref.)	1.02 (0.86–1.20)	0.838	1 (ref.)	1.02 (0.87–1.19)	0.840
Fish (excl. fried) ≥ 2/week	1 (ref.)	1.09 (0.93–1.27)	0.289	1 (ref.)	1.09 (0.93–1.28)	0.261	1 (ref.)	1.09 (0.94–1.26)	0.264
Dairy ≥ 3/day	1 (ref.)	0.70 (0.53–0.92)	0.011	1 (ref.)	0.72 (0.55–0.95)	0.021	1 (ref.)	0.71 (0.54–0.92)	0.011
At least 3 guidelines †	1 (ref.)	0.82 (0.69–0.96)	0.016	1 (ref.)	0.84 (0.71–0.99)	0.037	1 (ref.)	0.90 (0.77–1.05)	0.174
At least 3 guidelines ††	1 (ref.)	0.84 (0.70–0.99)	0.043	1 (ref.)	0.84 (0.70–0.99)	0.045	1 (ref.)	0.86 (0.73–1.02)	0.076

^†^, all fish; ††, excluding fried fish. AHEI, alternative healthy eating index; MUFA, monounsaturated fatty acids; PUFA, polyunsaturated fatty acids; SFA, saturated fatty acids; TEI, total energy intake; US, ultrasound. Statistical analysis conducted using logistic regression for categorical variables and analysis of variance for continuous variables; results were expressed as multivariable-adjusted odds ratio and (95% confidence interval) for the logistic models and as multivariable-adjusted means ± standard errors for the analysis of variance models. Models were adjusted for age groups (50–59/60–69/70–79), weight (continuous), educational level (high/middle/low), and smoking (former/never/current).

Further analysis using the energy regression method are presented in supplementary table 2. On bivariate analysis, participants in the highest tertiles of SI, BUA and SOS had a significantly higher consumption of MUFA (*p* < 0.05) than participants in the lowest tertiles. Participants in the highest tertiles of SI and BUA had a lower consumption of carbohydrates (*p* < 0.05), but a higher consumption of total fats (*p* < 0.05) and PUFA (*p* < 0.05). On multivariable analysis, participants in the highest tertiles of SI and BUA consumed significantly higher amounts of MUFA (*p* < 0.05).

## Discussion

The community-dwelling postmenopausal women of the OsteoLaus cohort with higher QUS parameters values – in the highest tertiles – was younger, of higher weight and BMI, consumed more MUFA, and less dairy and calcium than participants in the lowest tertiles. No differences were found regarding dietary patterns, dietary scores or compliance to dietary recommendations between the highest and the lowest tertiles of all QUS parameters.

### Characteristics of participants

In our study, 52.6% of the participants were overweight or obese. Participants in the highest tertiles of SI and BUA had higher weight and BMI.

Our findings are consistent with various studies of different cultural contexts. Similar studies conducted in the United Arab Emirates^32^ report high BMI as a predictive factor for high QUS parameters values; a study led in Germany found BMI, body mass and fat mass positively affected SOS, BUA and SI^33^; a study in China showed weight was a major determinant of QUS in men and women^34^ and in Greece, BMI mainly affected BUA^35^. In fact, higher BMI seem to be beneficial to bone QUS features for the mechanical effect of weight on bone itself; and the endocrinological effect of fat mass, given the conversion of androgens into estrogens happens in adipose tissue, even after menopause in women^36^. Moreover, in the Camargo cohort study, women with metabolic syndrome had higher QUS parameters than those without metabolic syndrome. Even though, after correction for BMI, this association disappeared, and out of the five single components of metabolic syndrome, only waist circumference was significantly associated^37^, the link between metabolic features and QUS parameters seemed to be evident.

### Fatty acids and QUS parameters

Participants in the highest tertiles of QUS parameters also consumed more total fats, where MUFA had strong significant association (*p* < 0.001), which remained after adjustment. This positive association does not exist with the “fatty and sugary” dietary score, as this pattern refers to saturated fat consumption. Menzel et al. compared bone health between 72 vegan and omnivore participants in a cross-sectional setting and showed that vegans had lower QUS values, and lower n-3 fatty (omega-3) acids levels, hallmark for polyunsaturated fats^38^. Similarly, in a longitudinal study of 118 Inuit women on a marine foods-based diet, consumers of large amounts of polyunsaturated fats^39^, the omega-3 polyunsaturated foods were positively associated with BUA measured at a 2-year follow-up. Unsaturated foods induce an anti-inflammatory effect on bone, through regulation of certain cytokines, decreasing bone resorption, and increasing calcium intestinal absorption – which has been tested in vitro^40^. Even though the consumption of fatty acids is not suggested as the priority nutrition recommendation for osteoporosis, the positive impact these foods have on this condition is known – some systematic reviews support this postulate with strong evidence^41,42^. Globally, the association between unsaturated fat consumption and osteoporosis is well known, particularly with BMD and less with osteoporotic fracture, and the trend seems similar for QUS.

### Calcium, vitamin D, dairy and QUS parameters

In our study, participants in the highest tertiles of SI or SOS showed lower calcium intake; and participants in the highest tertiles of SI, SOS or BUA were associated with lower dairy daily recommended portions according to SSN guidelines than participants in the lowest tertiles. Vitamin D failed to show any significant association with QUS parameters in our multivariable analysis. Yet, the vitamin D intake was estimated through the food questionnaire, but not assessed through a serum 25-hydroxyvitamin D dosage, thus not reflecting the exact supplementation.

Similar results were found in this Greek prospective study^43^ where QUS parameters were not changed in the dairy 12-month intervention group, receiving approximately 1200 mg calcium and 7.5 mg vitamin D3 through daily fortified dairy products, although significant improvement was observed in pelvis, total spine and total body BMD. In this Australian study^44^, calcium, folate and vitamin D3-fortified milk supplemented on a 6 month intervention period did not change QUS parameters. Devine et al.^45^ report that a high calcium supplementation itself – superior to 1053 mg a day—was not correlated with higher QUS parameters. Interestingly, high calcium supplementation combined with high physical activity – considered superior to 169 kcal/day – was significantly correlated with higher QUS values, 0.3% higher SOS and 3.6% higher SI values. The protective effect of calcium supplementation^10,12,13,46^ on bone health is well-established. Therefore, these findings suggest QUS might not be sensitive enough to detect the bone changes occurred at the amount of calcium evaluated in the above-mentioned studies.

Longitudinal intervention studies to observe calcium and vitamin D supplementation intake induced changes in bone as assessed by QUS in longer time periods are needed. However, QUS is currently not recommended for the monitoring of anti-osteoporotic therapy response, given the high variability between the QUS machines and insufficient clinical trials data^47^. QUS might be an efficient osteoporosis screening tool, rather than a monitoring tool^4^.

### Strength and limitations

To date, this is the largest study observing the relation between dietary characteristics and bone health as assessed by QUS parameters, in a homogenous Caucasian population. All nutritional assessments were based on standardized tools that had been previously tested and validated in the French-speaking population of Switzerland.

Our study has as limitation to be of cross-sectional nature, not allowing us to speculate any cause-effect relation. The included participants differed from the excluded participants as they were younger, of lower BMI and presented less frequently with diabetes. Dietary intake was assessed for the 4 weeks prior the study visit and not throughout the year or lifetime. In this study, we opted for the 0.05 threshold to define statistical significance as we tried to identify the largest number of dietary markers potentially associated with bone ultrasound parameters. Had a correction for multiple testing been applied such as the Bonferroni method, then the threshold would have been dependent on the number of dietary markers analysed and studies assessing fewer dietary markers would increase their likelihood of reporting statistically significant findings, while studies assessing a large array of dietary markers would be penalized. Indeed, applying a Bonferroni correction using the number of dietary markers studied led to a p-value threshold of 0.05/35 = 0.0014, thus cancelling most of the associations observed in this study.

## Conclusion

In the OsteoLaus cohort, postmenopausal women in the highest tertiles of QUS values were younger, of higher weight and BMI, consumed more monounsaturated fatty acids and less dairy and calcium than women in the lowest tertiles. No differences were found between QUS tertiles regarding dietary patterns. Dietary intake shows little influence on QUS parameters. Prospective studies would be needed to properly establish the impact of dietary characteristics on bone health assessed by QUS.

### Supplementary Information

Supplementary Information.

## Data Availability

The OsteoLaus and CoLaus|PsyColaus data used in this study cannot be fully shared as they contain potentially sensitive participant information. As discussed with the competent authority, the Research Ethic Committee of the Canton of Vaud, transferring or directly sharing this data would be a violation of the Swiss legislation aiming to protect the personal rights of participants. Non-identifiable, individual-level data are available for interested researchers, who meet the criteria for access to confidential data sharing, from the CoLaus Datacenter (CHUV, Lausanne, Switzerland). Instructions for gaining access to the CoLaus data used in this study are available at https://www.colaus-psycolaus.ch/professionals/how-to-collaborate/. Please fill in the Data Transfer Agreement form and indicate Prof. Marques-Vidal as the Provider.
