# Supplementary material for: Relationship between dietary characteristics and heel quantitative ultrasound parameters in postmenopausal women from the OsteoLaus cohort
Source: Sci Rep. 2024 Jan 18;14:1638. doi: 10.1038/s41598-024-51774-5 (PMC10796405; doi:10.1038/s41598-024-51774-5)
Supplement: Supplementary file 1 — Supplementary Information. [file 41598_2024_51774_MOESM1_ESM.pdf]

Supplementary table 1: Clinical characteristics of included and excluded participants, OsteoLaus study, Lausanne, Switzerland.

	Included	Excluded	P-value
Sample size	1203	272	
Age (years)	64.3 ± 7.5	65.7 ± 8.0	0.004
Age groups (%)			0.032
50-59	360 (29.9)	64 (23.5)	
60-69	562 (46.7)	127 (46.7)	
70-79	281 (23.4)	81 (29.8)	
Education (%)			0.057
High	168 (14.0)	29 (11.0)	
Middle	324 (26.9)	58 (22.0)	
Low	711 (59.1)	177 (67.0)	
BMI (kg/m^2)	25.7 ± 4.4	26.8 ± 4.9	<0.001
BMI categories (%)			0.006
Normal	570 (47.4)	106 (39.1)	
Overweight	430 (35.7)	99 (36.5)	
Obese	203 (16.9)	66 (24.4)	
Smoking status (%)			0.155
Never	557 (46.3)	118 (47.8)	
Former	446 (37.1)	78 (31.6)	
Current	200 (16.6)	51 (20.7)	
Physical activity: sedentary (%)	781 (64.9)	42 (76.4)	0.081
Participants with diabetes (%)	72 (6.0)	37 (14.2)	<0.001

BMI, body mass index. Results are expressed as number of participants (percentage) for categorical variables or as average ± standard deviation for continuous variables. Between group comparisons using chi-square for categorical variables or student's t-test for continuous variables.

Supplementary table 2 : Bivariate and multivariable analysis of the macronutrient intake in the lowest and highest tertiles of QUS parameters, OsteoLaus study, Lausanne, Switzerland.

	**Stiffness Index**			**Broadband US Attenuation**			**Speed of Sound**		
	**Lowest tertile**	**Highest tertile**	**P-value**	**Lowest tertile**	**Highest tertile**	**P-value**	**Lowest tertile**	**Highest tertile**	**P-value**
**Bivariate**									
Protein	-0.2 ± 11.8	0.3 ± 12.0	0.537	-0.5 ± 12.5	0.6 ± 12.5	0.231	0 ± 12.6	-0.4 ± 11.5	0.699
Vegetal protein	0.1 ± 5.5	-0.2 ± 4.9	0.430	0.2 ± 5.5	-0.5 ± 5.0	0.057	-0.1 ± 5.4	-0.2 ± 4.9	0.717
Animal protein	-0.3 ± 13.4	0.5 ± 13.5	0.394	-0.7 ± 14.2	1.1 ± 14.0	0.077	0 ± 14.1	-0.2 ± 13.0	0.837
Total carbohydrates	2.2 ± 40.1	-3.5 ± 36.6	0.036	3.3 ± 38.2	-4.1 ± 38.9	0.007	0.7 ± 40.6	-1.6 ± 37.1	0.405
Monosaccharides	2.4 ± 40.0	-1.7 ± 37.8	0.134	2.2 ± 38.1	-1.4 ± 38.3	0.192	1.5 ± 40.0	0 ± 38.0	0.604
Polysaccharides	-0.1 ± 35.9	-1.8 ± 31.4	0.496	1.1 ± 35.8	-2.7 ± 32.3	0.114	-0.8 ± 34.4	-1.6 ± 30.9	0.709
Total fat	-1.1 ± 14.1	1.4 ± 13.0	0.011	-1.2 ± 13.4	1.4 ± 13.5	0.007	-0.6 ± 14.0	0.8 ± 13.3	0.148
SFA	-0.1 ± 6.6	0.1 ± 6.1	0.642	-0.2 ± 6.5	0.2 ± 6.3	0.361	0 ± 6.4	-0.3 ± 5.9	0.459
MUFA	-0.7 ± 7.3	1.0 ± 7.4	0.001	-0.7 ± 7.0	0.9 ± 7.2	0.002	-0.5 ± 7.3	0.8 ± 7.5	0.013
PUFA	-0.3 ± 3.0	0.2 ± 2.9	0.024	-0.2 ± 2.8	0.3 ± 3.1	0.021	-0.2 ± 3.2	0.2 ± 3.0	0.104
Alcohol	1.8 ± 98.1	-0.1 ± 71.1	0.762	-0.2 ± 86.4	1.2 ± 72.7	0.804	3.6 ± 100.8	0.7 ± 73.1	0.642
**Multivariable**									
Protein	0.2 ± 0.6	-0.2 ± 0.6	0.697	0.1 ± 0.6	0 ± 0.7	0.956	0.1 ± 0.6	-0.5 ± 0.6	0.474
Vegetal protein	0.1 ± 0.3	-0.2 ± 0.3	0.478	0.1 ± 0.3	-0.4 ± 0.3	0.158	0.1 ± 0.3	-0.3 ± 0.3	0.307
Animal protein	0.1 ± 0.7	0 ± 0.7	0.947	-0.1 ± 0.7	0.5 ± 0.8	0.631	0 ± 0.7	-0.2 ± 0.7	0.803
Total carbohydrates	1.5 ± 2.0	-2.7 ± 2.0	0.158	2.0 ± 2.0	-2.8 ± 2.1	0.112	0.8 ± 2.0	-1.8 ± 2.0	0.365
Monosaccharides	2.0 ± 2.0	-1.3 ± 2.1	0.277	1.7 ± 2.0	-0.9 ± 2.1	0.392	1.2 ± 2.0	0.3 ± 2.0	0.767
Polysaccharides	-0.5 ± 1.7	-1.4 ± 1.8	0.714	0.3 ± 1.8	-1.9 ± 1.8	0.416	-0.3 ± 1.7	-2.1 ± 1.7	0.469
Total fat	-0.9 ± 0.7	1.1 ± 0.7	0.056	-0.9 ± 0.7	1.0 ± 0.7	0.076	-0.6 ± 0.7	0.7 ± 0.7	0.194
SFA	-0.2 ± 0.3	0.1 ± 0.3	0.542	-0.2 ± 0.3	0.2 ± 0.3	0.447	-0.1 ± 0.3	-0.2 ± 0.3	0.829
MUFA	-0.5 ± 0.4	0.8 ± 0.4	0.020	-0.5 ± 0.4	0.6 ± 0.4	0.041	-0.4 ± 0.4	0.7 ± 0.4	0.057
PUFA	-0.2 ± 0.2	0.2 ± 0.2	0.087	-0.2 ± 0.2	0.2 ± 0.2	0.112	-0.2 ± 0.2	0.2 ± 0.2	0.123
Alcohol	1.3 ± 4.4	0.5 ± 4.6	0.905	-0.4 ± 4.1	1.4 ± 4.3	0.784	2.3 ± 4.5	2.1 ± 4.5	0.965

MUFA, monounsaturated fatty acids; PUFA, polyunsaturated fatty acids; SFA, saturated fatty acids; US, ultrasound. Analysis conducted on the residuals after applying the energy adjustment regression method. Results are expressed in gr/day and as average ± standard deviation for bivariate analyses and as adjusted average ± standard error for multivariable analyses. Between group comparisons using student's t-test for bivariate analyses and analysis of variance adjusting for age groups (50-59/60-69/70-79), weight (continuous), educational level (high/middle/low), and smoking (former/never/current) for multivariable analyses.
